# Downregulation of PGC-1*α* Prevents the Beneficial Effect of EET-Heme Oxygenase-1 on Mitochondrial Integrity and Associated Metabolic Function in Obese Mice

**DOI:** 10.1155/2016/9039754

**Published:** 2016-12-20

**Authors:** Shailendra P. Singh, Lars Bellner, Luca Vanella, Jian Cao, John R. Falck, Attallah Kappas, Nader G. Abraham

**Affiliations:** ^1^New York Medical College, Departments of Medicine and Pharmacology, Valhalla, NY, USA; ^2^Department of Drug Science, Section of Biochemistry, University of Catania, Catania, Italy; ^3^First Geriatric Cardiology Division, Chinese PLA General Hospital, Beijing, China; ^4^Department of Biochemistry, University of Texas Southwestern Medical Center, Dallas, TX, USA; ^5^The Rockefeller University, New York, NY, USA

## Abstract

*Background/Objectives*. Obesity and metabolic syndrome and associated adiposity are a systemic condition characterized by increased mitochondrial dysfunction, inflammation, and inhibition of antioxidant genes, HO-1, and EETs levels. We postulate that EETs attenuate adiposity by stimulating mitochondrial function and induction of HO-1 via activation of PGC-1*α* in adipose and hepatic tissue.* Methods*. Cultured murine adipocytes and mice fed a high fat (HF) diet were used to assess the functional relationship among EETs, PGC-1*α*, HO-1, and mitochondrial signaling using an EET-agonist (EET-A) and PGC-1*α*-deficient cells and mice using lentiviral PGC-1*α*(sh).* Results*. EET-A is a potent inducer of PGC-1*α*, HO-1, mitochondrial biogenesis (cytochrome oxidase subunits 1 and 4 and SIRT3), fusion proteins (Mfn 1/2 and OPA1) and fission proteins (DRP1 and FIS1) (*p* < 0.05), fasting glucose, BW, and blood pressure. These beneficial effects were prevented by administration of lenti-PGC-1*α*(sh). EET-A administration prevented HF diet induced mitochondrial and dysfunction in adipose tissue and restored VO_2_ effects that were abrogated in PGC-1*α*-deficient mice.* Conclusion*. EET is identified as an upstream positive regulator of PGC-1*α* that leads to increased HO-1, decreased BW and fasting blood glucose and increased insulin receptor phosphorylation, that is, increased insulin sensitivity and mitochondrial integrity, and possible use of EET-agonist for treatment of obesity and metabolic syndrome.

## 1. Introduction

Obesity is a global epidemic and a major risk factor in the development of metabolic syndrome and diabetes associated complications such as cardiovascular disease, kidney disease, hypertension, and neuropathies [1–3]. Epoxyeicosatrienoic acids (EETs) are arachidonic acid derived metabolites generated by a family of cytochrome P450 (CYP) monooxygenases and epoxygenases [4, 5]. EETs are rapidly hydrolyzed by ROS and by soluble epoxide hydrolase (sEH) to their respective dihydroxyepoxytrienoic acids [6–8]. EET agonists prevent both adiposity and vascular complications both in vitro and in vivo and obesity-induced adipose tissue expansion impairs the CYP epoxygenase pathway and the generation of EET in vivo [9–12].

Mitochondrial biogenesis, oxygen consumption, and oxidative phosphorylation are regulated by peroxisome proliferator-activated receptor gamma coactivator 1-alpha (PGC-1*α*) [13], which in turn is regulated by posttranslational modifications, including phosphorylation and deacetylation by AMPK and SIRT1, respectively [14]. PGC-1*α* activates several key components of the adaptive thermogenesis program, including the stimulation of energy uptake, and mitochondrial fatty acid oxidation. Transgenic mice with mildly elevated muscle levels of PGC-1*α* are resistant to age-related obesity [15]. Furthermore, mice that are lacking PGC-1*α* in adipose tissue and fed HF diet develop insulin resistance and have increased circulating lipid levels [16]. Additionally, the uncoupling proteins 1–3 (UCP1–3) are located in the mitochondrial intramembranous space and play a key role in thermogenesis [17]. UCP-1 is highly expressed in brown adipose tissue [17] in a PGC-1-dependent manner to increase energy expenditure and oxygen consumption [17].

Heme oxygenase-1 (HO-1) is a stress response enzyme which in rodents and humans degrades heme to carbon monoxide, iron, and the potent antioxidant and anti-inflammatory molecule biliverdin, which is subsequently degraded to bilirubin [18, 19], thereby offering increased protection against obesity-induced ROS and hypertension [20]. A strong case has been made for the existence of a positive feedback loop between EET and HO-1. Sacerdoti et al. reported that EET-mediated vascular dilation is dependent on HO-1 expression and EETs increase HO-1 protein levels and HO activity in vitro [21, 22]. A decrease in HO-1 levels increases adipocyte hypertrophy contributing to elevated liver fat content and steatohepatitis that is associated with mitochondrial dysfunction.

The majority of cellular ROS that contributes to increased adipogenesis is generated by the mitochondria and contributes to energy metabolism [23]. One of the seven mammalian sirtuins, sirtuin 3 (SIRT3), a mitochondrial deacetylase, was recently reported to be the target of PGC-1*α* and impact mitochondrial processes, such as mitochondrial biogenesis, suppression of ROS, and energy metabolism [23], including mitochondrial fatty acid oxidation [24]. Mitochondrial energy and metabolic demands as well as viability are tightly linked to mitochondrial network morphology and depend greatly on quality control and a balanced relationship between mitochondrial fusion (the merge of dysfunctional to functional) and fission (budding and isolation of dysfunctional mitochondria) processes. Mitochondrial fission is orchestrated by the dynamin-related protein 1 (DRP1) and the mitochondrial fission 1 (Fis1) protein [25, 26], while the fusion process is controlled by the autosomal dominant optic atrophy 1 (OPA1) protein, located on the mitochondrial inner membrane, together with the mitochondrial fusion proteins mitofusins 1 and 2 (Mfn 1 and 2), located on the mitochondrial outer membrane [27, 28]. Studies of the balance between fission and fusion have shown that development of obesity and insulin resistance is associated with a reduction in mitochondrial fusion [26, 29, 30] and increased mitochondrial fission [31].

Given the regulatory role of PGC-1*α* on adipogenesis and mitochondrial function, we hypothesize that the EET-mediated modulation of adiposity and the subsequent increase of mitochondrial fusion, oxidative phosphorylation, and HO-1 expression is dependent upon PGC-1*α*. Further, the effect of PGC-1*α*-deficiency on metabolic parameters and mitochondrial biogenesis, function, and fusion potential was performed using lentiviral gene delivery that is effective for 9 months [32].

## 2. Materials and Methods

### 2.1. Animal Experimentation and Generation of Lentiviral Vector-Mediated PGC-1*α* Deficient Mice

All animal experiments followed a PLA, General Hospital, Beijing, China, and NYMC IACUC institutionally approved protocol in accordance with the NIH Guidelines. Male C57bl6 mice were used in the studies. Two separate experiments (A and B) were performed. In the first experiment (A) we investigated the effect of PGC-1*α* ablation and* short-term* EET-A treatment of mice fed a HF diet for 8 weeks. In the second experiment (B) we examined the effect of an EET-A regimen in mice fed a HF diet for 24 weeks: for experiment (A) with PGC-1*α*-deficient mice, animals were divided into 4 groups: (1) lean, (2) HFD, (3) HFD+EET-analog [EET-A is sodium (S,Z)-2-(13-(3-pentylureido)tridec-8-enamido)succinate], and (4) HFD+EET-A+PGC-1*α* lentivirus. Lean mice (group 1) were fed ad libitum a normal chow diet containing 11% fat, 62% carbohydrate, and 27.0% protein with total calories of 12.6 KJ/g. The remaining animals (groups 2, 3, and 4) were fed a HF diet containing 58% fat (from lard), 25.6% carbohydrate, and 16.4% protein with total calories of 23.4 KJ/g (Harlan, Teklad Lab Animal Diets, US) for 8 weeks. Mice were treated as follows: group (1) was fed normal chow diet, group (2) was fed HF diet, group (3) was injected with EET-A, intraperitoneally, every other day for 4 weeks at a dose of 1.5 mg/100 gm of body weight, and group (4) received a 2-bolus injection of PGC-1*α*(sh) lentivirus (Dharmacon, Lafayette, CO) injected into the retroorbital vein at a concentration of 40–70 *∗* 10^8^ TU/mouse in 80–100 *µ*l and received EET-A injections identical to group 3.

For the second experiment (long-term effect of EET-agonist) (B) mice were divided into three groups similar to groups 1, 2, and 3 in experiment (A) but were maintained on the diets for 24 weeks and group (3) received injections with EET-A every other day for the last 8 weeks of the experiment.

For fasting blood glucose level measurement mice were fasted for 6 h with access to water, after blood was obtained from tail and blood glucose concentration was determined with the Accuchek Advantage glucometer (Roche, Madison, WI). At the end of the experimental period, mice were anesthetized with sodium pentobarbital (65 mg/Kg, i.p.) and, at the time of sacrifice, body weight was measured.

### 2.2. Cell Culture

3T3-L1 murine preadipocytes were purchased from ATCC (ATCC, Manassas, VA). After thawing, 3T3-L1 cells were cultured at 37°C in a 5% CO_2_ incubator in *α*-minimal essential medium (*α*-MEM, Invitrogen, Carlsbad CA) supplemented with 10% heat inactivated fetal bovine serum (FBS, Invitrogen, Carlsbad, CA) and 1% antibiotic/antimycotic solution (Invitrogen, Carlsbad, CA). The medium was changed after 48 h and every 3-4 days thereafter as described previously [10]. For adipogenesis studies the medium was replaced with adipogenic medium (Dulbecco's modified Eagle Medium (DMEM)) with high glucose (Invitrogen), supplemented with 10% (v/v) FBS, 10 *μ*g/ml insulin (Sigma-Aldrich, St. Louis, MO), 0.5 mM dexamethasone (Sigma-Aldrich), and 0.1 mM indomethacin (Sigma-Aldrich) and the cells were cultured for an additional 8 days. Cells were cultured in the absence and presence of EET-A at a dose of 10 *μ*M. At the experimental endpoints, cells were collected by trypsinization, washed once with PBS, and then lysed for protein measurements and for RNA extraction.

### 2.3. Generation of Lentiviral Vector-Mediated PGC-1*α* Deficient 3T3-L1 Derived Adipocytes

SMART vector lentiviral shRNA-PPARGC1A or scrambled RNA (Dharmacon, Lafayette, CO) was applied to 3T3-L1 cells to establish a stably transduced cell line. Briefly, 1 × 10^6^ cells were seeded in 6-well plates 1 day prior to transduction. On the day of transduction, the transduction medium was made by 1 × 10^6^ transducing units (TU) of lentiviral particles with 0.5 ml *α*-MEM growth medium being applied to each well and incubated for 3 h to maximize the contact between each cell and lentiviral particles. Cells were also treated with the transduction medium without lentiviral particles which served as untransduced control. Growth medium (1.5 ml) was then added to each well in the presence of 8 *μ*g/ml polybrene (final concentration). After 48 h incubation, antibiotic selection medium (*α*-MEM growth medium with 10 *μ*g/ml puromycin) was used to kill all the untransduced cells. Cells were then cultured and maintained as outlined above.

### 2.4. RNA Extraction and Real-Time PCR

Total RNA was extracted from 3T3 cells using TRIzol® (Ambion, Austin, TX) and from frozen adipose tissue by RNeasy® Lipid Tissue (Qiagen), as per instructions provided by the manufacturers. RNA was determined by measuring the absorbance at 260 nm (A260) with a Biotek™ plate reader and the Take3™ plate (Biotek, Winooski, VT), and assessed by the A260/A280 ratio. cDNA was synthesized from total RNA using the High Capacity cDNA Reverse Transcription Kit (Applied Biosystems), after real-time PCR was performed using TaqMan® Fast Universal Master Mix (2x), on a 7500 HT Fast Real-Time PCR System (Applied Biosystems). Specific TaqMan Gene Expression Assays probes for mouse HO-1, PGC1*α*, UCP1, COX-IV (cytochrome c oxidase subunit-IV), adiponectin, TNF*α*, IL-6, Mfn1, Mfn2, Drp1, Fis1, OPA1, aP2, SIRT3, C/EBP*α*, and GAPDH were used as previously described [33].

### 2.5. Western Blot Analysis

Frozen mouse adipose tissue was ground under liquid nitrogen and suspended in homogenization buffer (mmol/l: 10 phosphate buffer, 250 sucrose, 1.0 EDTA, 0.1 PMSF, and 0.1% v/v tergitol, pH 7.5). For in vitro western blot analysis pelleted cells were lysed with lysis buffer supplemented with protease and phosphatase inhibitors (cOmplete™ Mini and PhosSTOP™, Roche Diagnostics, Indianapolis, IA). Immunoblotting for HO-1, SIRT1, UCP1, TFAM, aP2, PEG1/MEST, MnSOD, AMPK, pAMPK, AKT, pAKT, FAS, and PGC-1*α* and phosphorylation of insulin receptors (IR) IRp972, IRp1146, COX-IV, adiponectin, *β*-actin, and GAPDH were performed as previously described [8, 34].

### 2.6. Oxygen Consumption

Oxygen consumption (VO_2_) and carbon dioxide production (VCO_2_) were measured using the Oxylet gas analyzer and air flow unit (Panlab-Bioseb, Vitrolles, France). Animals were allowed to acclimatize in the oxygen consumption chambers for 2 hours three times a week for 3 weeks prior to the recording of data. Mice were placed in the Oxylet chamber and the flow rate was adjusted and the dCO2 maintained between 0.4 and 0.8 as per the instructions provided by the manufacturer, after the hourly respiratory quotients were calculated based on the VCO_2_ and VO_2_ obtained by the gas analyzer. Individual readings were recorded twice per mouse. The data for VO_2_ are expressed as the consumed volume of oxygen per kilogram body weight per minute (ml/kg/min). The respiratory quota is expressed as CO_2_ eliminated divided by the O_2_ consumed.

### 2.7. Statistical Analysis

Data are expressed as means ± SEM. Significance of difference in mean values was determined using one-way analysis of variance followed by the Bonferroni posttest for comparison between groups. *p* < 0.05 was considered to be significant.

## 3. Results

### 3.1. Effect of EET-A on Mitochondrial Fission (DRP1 and Fis1) and Fusion (Mfn1 and Mfn2) in Wildtype and Lenti shRNA Vector-Mediated PGC-1*α* Deficient 3T3-L1-Derived Adipocytes

**Figure 1:**
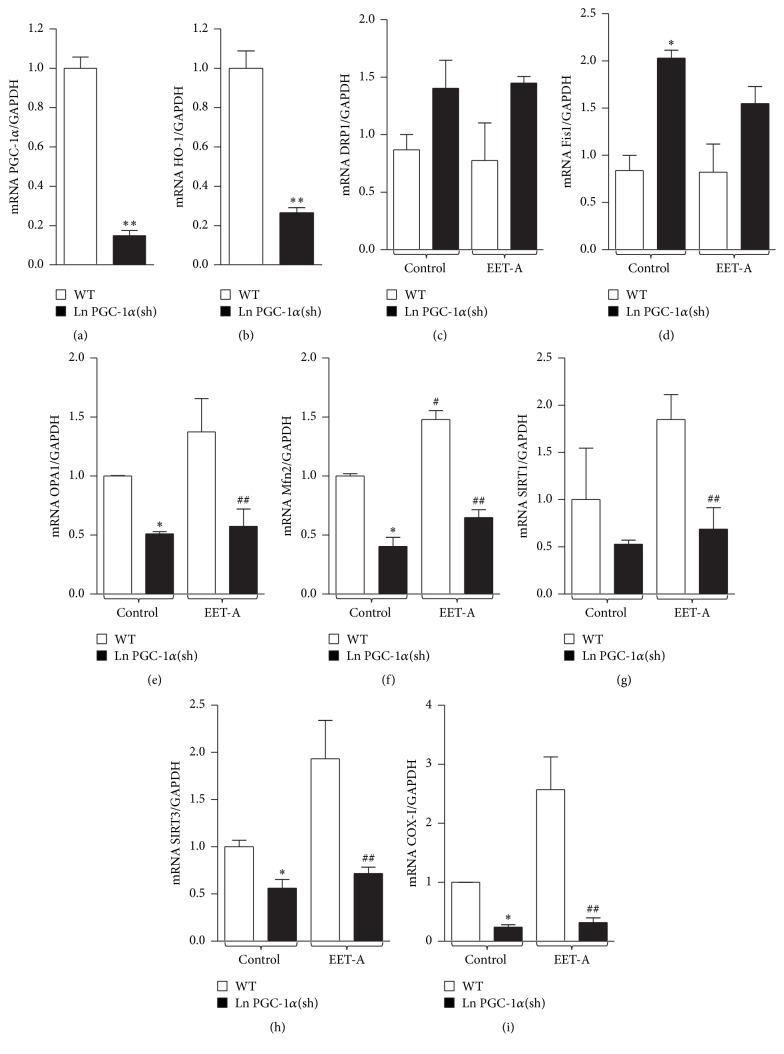
Effect of EET-A treatment on WT and PGC-1*α*-deficient 3T3-L1 adipocytes cells on gene expression related to mitochondrial biogenesis and dynamics. mRNA expression of PGC-1*α* (a), HO-1 (b) in WT and PGC-1*α* deficient 3T3-L1-derived adipocyte cells, and DRP1 (c), Fis1 (d), OPA1 (e), Mfn2 (f), SIRT1 (g), SIRT3 (h), and COX-I (i) in WT and PGC-1*α* deficient 3T3-L1-derived adipocyte cells in the absence and presence of EET-A (10 *μ*M) treatment. Results are mean ± SE, *n* = 3-4, ^*∗*^ *p* < 0.05 versus WT, ^*∗∗*^ *p* < 0.01 versus WT, ^#^ *p* < 0.05 versus WT, and ^##^ *p* < 0.05 versus WT-EET-A. White bars: WT; black bars: PGC-1*α* deficient cells.

To examine whether EET-A can modulate the mitochondrial fusion-to-fission ratio through PGC-1*α* we knocked down PGC-1*α* in 3T3-L1 adipocyte cells using lentivirus vector carrying target specific shRNA. RT-PCR data show more than 80% inhibition of PGC1*α* mRNA levels after transduction with PGC-1*α* shRNA (Figure 1(a)) and a concomitant decrease in HO-1 mRNA expression (Figure 1(b)). PGC-1*α* knockdown resulted in increased mRNA expression of Fis1 (*p* < 0.05) as compared to WT cells. The mRNA expression of DRP1 was not affected by PGC-1*α* silencing. While EET-A did not affect fission related expression in WT cells (Figures 1(c) and 1(d)), the Mfn2 levels were elevated (*p* < 0.05) in WT cells treated with EET-A as compared to WT control cells. The mRNA levels of OPA1 were not increased by EET-A (Figure 1(e)). PGC-1*α* silencing decreased (*p* < 0.05) Mfn2 and OPA1 mRNA expression as compared to WT control cells. Importantly, EET-A had no effect on Mfn2 or OPA1 mRNA expression levels in PGC-1*α* deficient cells (Figures 1(e) and 1(f)).

The mRNA expression levels of SIRT3 and COX-I were decreased (*p* < 0.05) in the PGC-1*α* knockdown cells as compared to WT cells. While EET treatment had only moderate effect on the expression of SIRT1, SIRT3, and COX-I in WT cells, the expression of these genes were not affected by EET-A treatment of PGC-1*α* deficient cells (Figures 1(g), 1(h), and 1(i)).

### 3.2. Effect of Short- versus Long-Term EET-A on Body Weight, Blood Pressure, Fasting Blood Glucose, Oxygen Consumption, and Respiratory Quotient 

**Figure 2:**
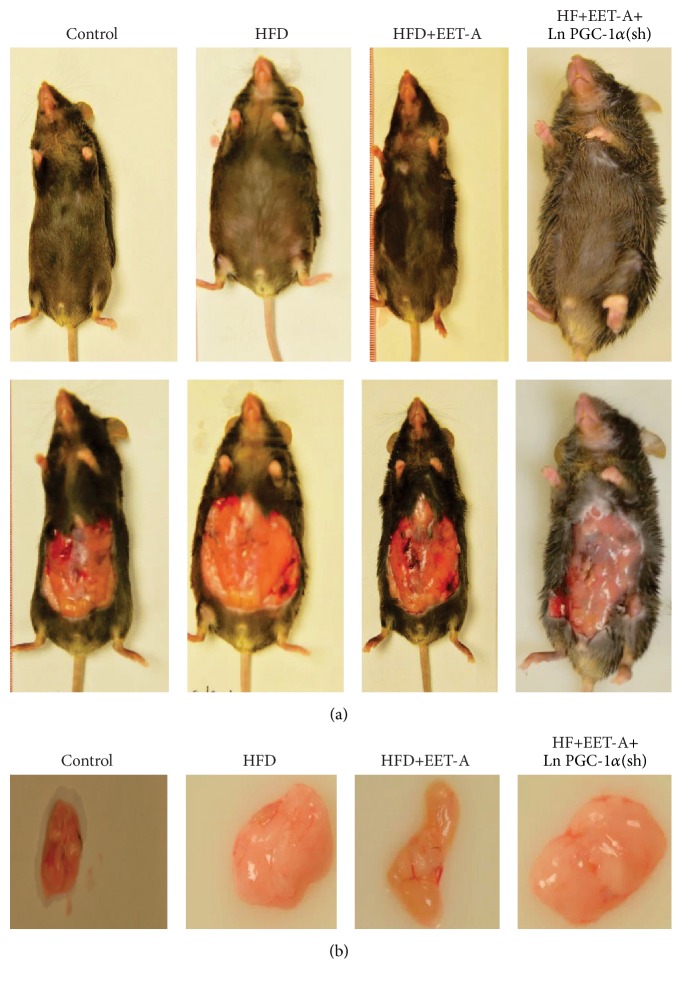
Appearance of (a) body and (b) fat size of mice during a period of 8 weeks on high fat diet.

We examined the effect of EET-A on body weight, blood pressure, fasting blood glucose, O_2_ consumption, and the ratio of CO_2_/O_2_ in mice fed a HF diet for 8 and 24 weeks (Figure 2). As expected, mice on a HF diet displayed a decrease (*p* < 0.05) in VO_2_ consumption. However, HF diet fed mice treated with EET-A exhibited an increase (*p* < 0.05) in oxygen consumption with concomitant lowering of VCO_2_/VO_2_. Importantly, EET-A had no effect on oxygen consumption or respiratory quotient in HF diet fed PGC-1*α* deficient mice (Table 1). Fasting blood glucose levels and blood pressure were increased in mice fed a HF diet as compared to control animals (Table 1, *p* < 0.05). Importantly, fasting blood glucose levels were normalized in mice fed a HF diet and treated with EET-A (*p* < 0.01) when compared to lean mice. The EET-A mediated decrease in fasting blood glucose levels was prevented in PGC-1*α* deficient animals fed a HF diet. Importantly, regardless of the duration, EET-A treatment normalized all four metabolic parameters (Table 1).

### 3.3. Effect of Short-Term EET-A on the Expression of Mitochondrial Fission (DRP1 and Fis1) and Fusion (Mfn1, Mfn2) in Adipose Tissue of High Fat Diet Fed Control and PGC-1*α* Deficient Mice

**Figure 3:**
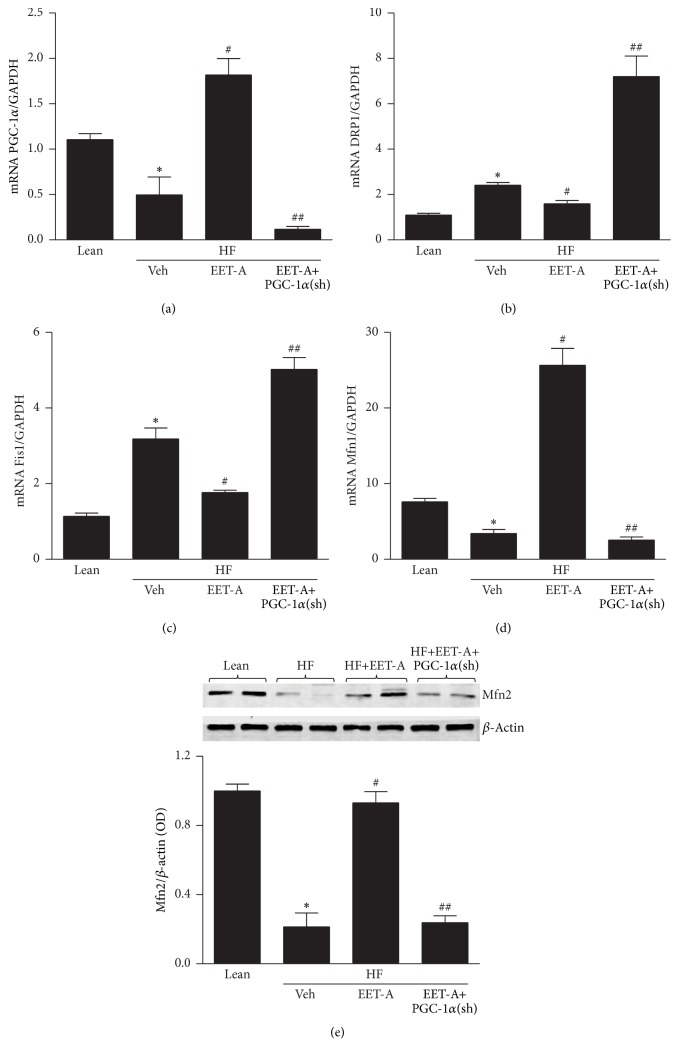
Effect of short-term EET-A treatment on the expression of genes related to mitochondrial biogenesis and dynamics in the adipose tissue of mice. mRNA expression of PGC-1*α* (a), Drp1 (b), Fis1 (c), and Mfn1 (d) and protein expression of Mfn2 (e) in the adipose tissues of lean, HF diet fed (HF), HF diet EET-A-treated (EET-A), and PGC-1*α*-deficient (Ln shPGC-1*α*) mice. Results are mean ± SE, *n* = 3-4, ^*∗*^ *p* < 0.05 versus lean, ^#^ *p* < 0.05 versus HF, and ^##^ *p* < 0.05 versus HF+EET.

PGC-1*α* mRNA expression in HF diet fed mice treated with vehicle (Veh) solution (H_2_O) was reduced (*p* < 0.05) as compared to lean mice. Importantly EET-A treatment nullified the inhibitory effect of HF diet on PGC-1*α* expression. EET-A treatment of PGC-1*α*-deficient mice had no effect on PGC-1*α* mRNA expression (Figure 3(a)). The mRNA expression levels of mitochondrial fission related DRP1 and Fis1 in adipose tissue were increased (*p* < 0.05) by a HF diet. Interestingly, EET-A treatment of mice on a HF diet decreased (*p* < 0.05) the adipose tissue levels of both DRP1 and Fis1 as compared to HF diet fed mice. Inversely, DRP1 and Fis1 mRNA levels VAT of PGC1*α*-deficient mice were increased (*p* < 0.05) as compared to HF diet fed mice treated with EET-A alone (Figures 3(b) and 3(c)). The expression levels of fusion related Mfn1 and Mfn2 were significantly decreased by a HF diet in adipose tissue. Prominently, EET-A treatment of mice on a HF diet increased (*p* < 0.05) the expression levels of both Mfn1 and Mfn2 as compared to HF diet fed mice (Figures 3(d) and 3(e)). The observed increase was PGC-1*α*-dependent as the levels of both Mfn1 and Mfn2 in adipose tissue from PGC1*α*-deficient mice reduced (*p* < 0.05) as compared to HF diet fed mice treated with EET-A alone (Figures 3(d) and 3(e)).

### 3.4. Effect of Long-Term EET-A on Insulin Receptor Phosphorylation and pAMPK and pAKT Levels in Adipose Tissue of Mice Fed a HF Diet

**Figure 4:**
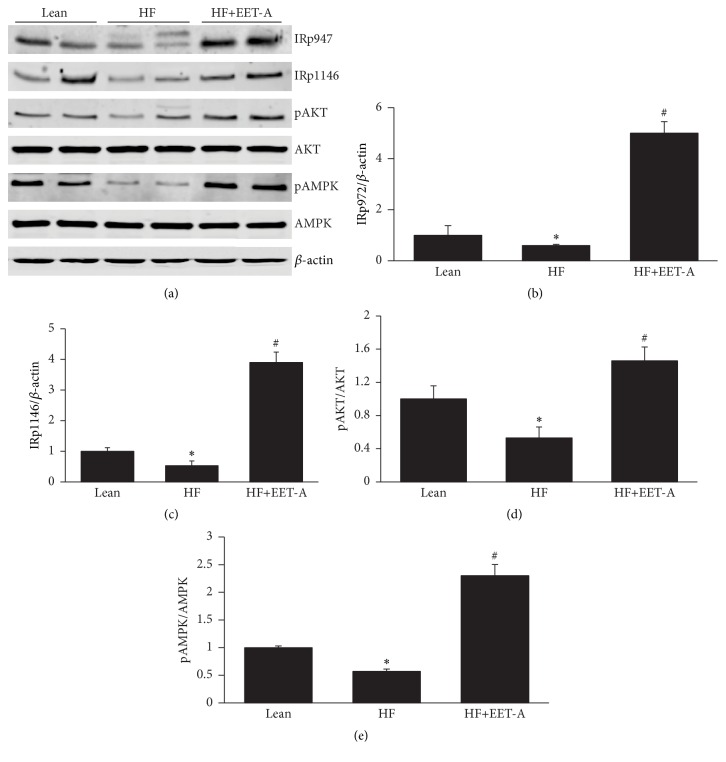
Effect of short-term EET-A treatment on insulin receptor phosphorylation and AKT and AMPK signaling in adipose tissue of mice fed HF diet. (a) Representative western blots and ((b) to (f)) densitometry analyses of WBs in adipose tissue of mice; (b) insulin receptor phosphorylation IRp-Tyr-972; (c) insulin receptor phosphorylation IRp-Tyr-1146; (d) phosphorylated protein kinase B (pAKT); (e) phosphorylated adenosine monophosphate protein kinase (pAMPK) proteins in mice fed a normal diet lean and mice fed a high fat (HF) diet treated with EET-A. Results are mean ± SE, *n* = 4, ^*∗*^ *p* < 0.05 versus lean, and ^#^ *p* < 0.05 versus HF.

Mice fed a HF diet had a decrease (*p* < 0.05) in the protein expression of IRp-Tyr 972, IRp-Tyr 1146, pAMPK, and pAKT. Mice fed a HF diet and treated with EET-A displayed an 8.2-fold increase (*p* < 0.001) in IRp-Tyr 972 and a 7.3-fold increase in IRp-Tyr 1146 (*p* < 0.01) as compared to mice fed a HF diet alone (Figure 4). Likewise, EET-A increased the pAKT/AKT and pAMPK/AMPK ratio (*p* < 0.05) when compared to mice fed a HF diet alone (Figure 4).

### 3.5. Effect of EET-A on Induction of HO-1, PGC1-*α*, SIRT1, and Adiponectin Expression in Adipose Tissue of Mice Fed a HF Diet

**Figure 5:**
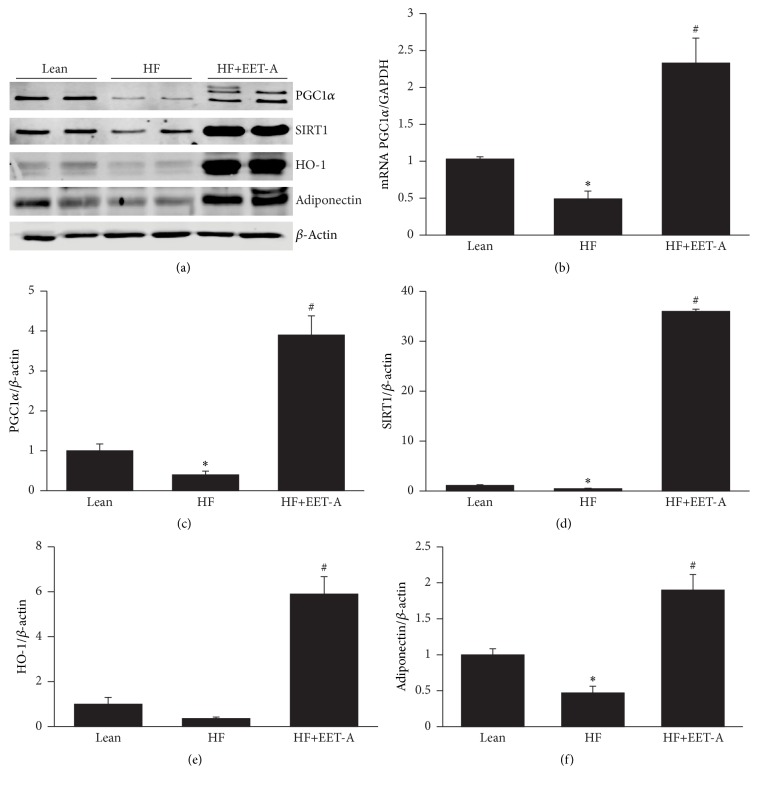
Effect of long-term EET-A treatment on the expression of PGC-1*α*, SIRT1, HO-1, and adiponectin in adipose tissue of mice fed HF diet. (a) Representative Western blots, (b) protein, and (c) mRNA levels of PGC-1*α*. Protein levels of (d) SIRT1, (e) HO-1, and (f) adiponectin. Data are normalized to *β*-actin for protein or GAPDH for mRNA. Results are mean ± SE, *n* = 4, ^*∗*^ *p* < 0.05 versus lean, and ^#^ *p* < 0.05 versus HF.

Western blot analysis demonstrated that mice fed a HF diet for 24 weeks had decreased levels of HO-1 protein expression (Figure 5) as compared to mice fed a normal chow diet. Treatment with EET-A for 8 weeks resulted in a 16-fold increase (*p* < 0.01) in HO-1 protein expression as compared to mice fed a HF diet alone (Figures 5(a) and 5(e)). Visceral adipose tissue obtained from mice fed a HF exhibited a decrease (*p* < 0.05) in PGC-1*α*, SIRT1, and adiponectin expression as compared to lean mice fed a regular chow diet, while EET-A increased (*p* < 0.05) PGC-1*α*, SIRT1, and adiponectin expression levels as compared to the mice fed a HF diet alone (Figure 5).

### 3.6. Effect of EET-A on TFAM, UCP1, and MnSOD Levels in Adipose Tissue of Mice Fed a HF Diet

**Figure 6:**
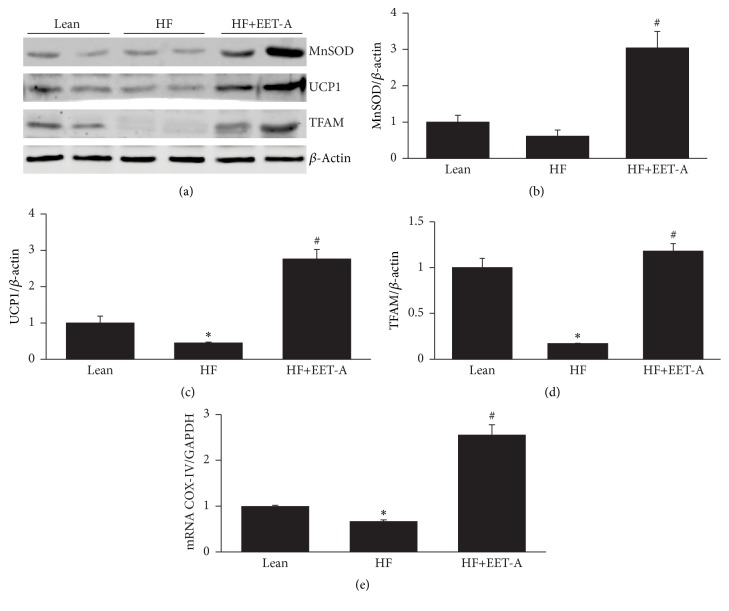
Effect of long-term EET-A treatment on the expression of genes involved in mitochondrial biogenesis and dynamics in adipose tissue of mice fed HF diet. Representative Western blots (a). Densitometry analysis of MnSOD (b), UCP1 (c), TFAM, (d) protein levels, and COX-IV mRNA levels (e) in adipose tissues of lean, HF diet fed (HF), and HF diet EET-A-treated (EET-A) mice. Results are mean ± SE, *n* = 4, ^*∗*^ *p* < 0.05 versus lean, ^#^ *p* < 0.05 versus HF.

Our analysis demonstrated a decrease (*p* < 0.05) in the UCP1, TFAM, and MnSOD expression levels in adipose tissue of mice fed a HF diet as compared to lean mice fed a regular chow diet. EET-A increased (*p* < 0.05) MnSOD, UCP1, and TFAM expression as compared to mice fed a HF diet alone (Figure 6). mRNA expression of COX-IV was elevated in obese mice treated with EET- A (group 3) (*p* < 0.05) (Figure 6). We examined the effect of EET-A treatment on body and fat appearance. EET-A-treatment visibly reduced weight gain in mice fed HFD (Figure 2(a)), an effect that was prevented in PGC-1*α*-deficient mice. The final weights after 4 or 8 weeks of vehicle or EET-A treatment are presented in Table 1. The EET-A-mediated reduction of weight gain was observed as early as after 4 weeks. As seen in Figure 2(b), visceral fat in control mice fed HFD was decreased by EET-A-treatment, but this EET-A-mediated effect was prevented in PGC-1*α*-deficient mice.

### 3.7. EET-A Decreases Expression of Adipogenic and Inflammatory Mediators in Adipose Tissue of Mice Fed a HF Diet

**Figure 7:**
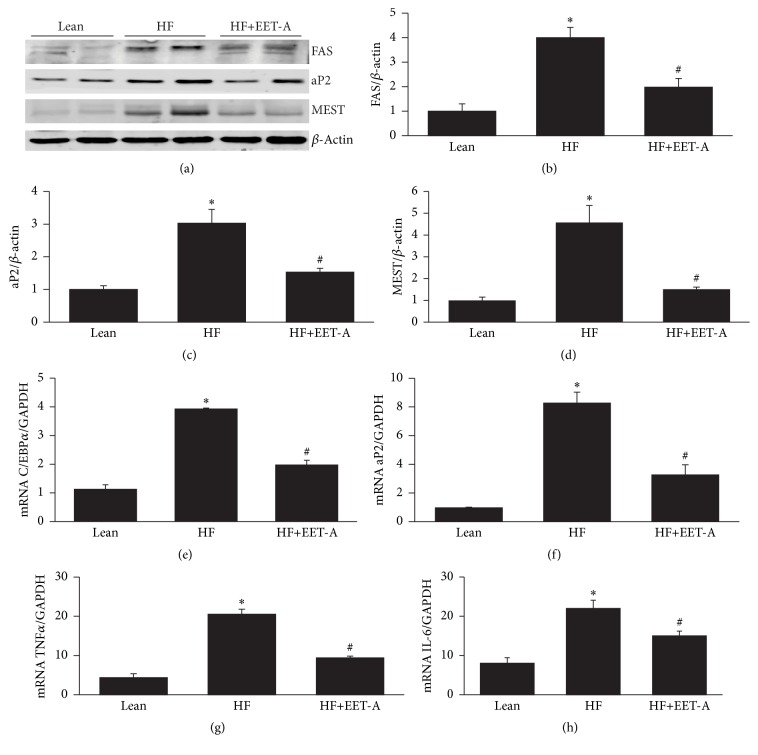
Effect of high fat diet and long-term treatment with EET-A on protein and mRNA expression of adipogenic markers and inflammatory cytokines. (a) Representative Western blots of FAS, aP2, and MEST proteins. Densitometric analyses of (b) FAS and (c) MEST proteins. (d) Protein and (e) mRNA levels of aP2. (f) C/EBP*α*, (g) TNF-*α*, and (h) IL-6 mRNA levels. Data are normalized to *β*-actin for protein or GAPDH for mRNA. Results are mean ± SE, *n* = 4, ^*∗*^ *p* < 0.05 versus lean, and ^#^ *p* < 0.05 versus lean-HF.

A HF diet increased (*p* < 0.05) the expression of adipogenic marker proteins FAS, aP2, and MEST (Figures 7(a)–7(d)) as compared to lean mice fed a regular chow diet. Mice treated with EET-A had decreased protein levels of FAS, MEST, and aP2 in adipose tissue as compared to mice fed a HF diet alone (Figures 7(a)–7(d)) (*p* < 0.05). A HF diet was associated with an increase (*p* < 0.05) in the adipose tissue expression of aP2 and C/EBP*α* mRNA levels as compared to lean mice fed a regular chow diet (Figures 7(e) and 7(f)). However, mice fed a HF diet and treated with EET-A exhibited decreased aP2 and C/EBP*α* mRNA levels in adipose tissue as compared to mice fed a HF diet (Figures 7(e) and 7(f)) (*p* < 0.05). Adipose tissue of mice fed a HF diet presented with an increase (*p* < 0.05) in the mRNA expression levels of the inflammatory mediators, TNF*α* and IL-6, in adipose tissue as compared to lean mice. Administration of EET-A attenuated (*p* < 0.01) the HF diet induced increase in TNF*α* and IL-6 levels in mouse adipose tissue (Figures 7(g) and 7(h)).

## 4. Discussion

This study demonstrates that EET participates in the regulation of mitochondrial function in adipocyte cells in vitro and in adipose and hepatic tissues in vivo by influencing the levels of PGC-1*α* and HO-1 expression. We show that EET is upstream of PGC-1*α* and that contributes to increased mitochondrial biogenesis, function, and fusion potential, leading to an improvement of metabolic parameters in obese mice that is PGC-1*α* dependent. EET was effective in the restoration of mitochondrial integrity in the early stage of obesity (8 weeks) as well as in chronic obesity (24 weeks). The following key findings substantiate this conclusion.

Firstly we show that lentiviral vector-mediated knockdown of PGC-1*α* cultured murine adipocytes in vitro increases the mitochondrial fission potential (DRP1 and Fis1) at the expense of mitochondrial quality control and fusion potential (OPA1, Mfn2) as well as reducing the expression of genes involved in oxidative phosphorylation and suppression of ROS (COX-I, SIRT1, and SIRT3). While treatment of WT adipocytes with EET-agonist had a beneficial effect on the mitochondrial function and fusion potential, these effects were absent in PGC-1*α*-deficient adipocytes.

In vivo, a HF diet precipitated increased expression of the genes regulating mitochondrial fission, while concomitantly reducing the expression of the genes responsible for mitochondrial quality control and fusion processes in visceral adipose tissue as compared to mice fed a regular chow diet. EET-agonist treatment of mice fed a HF diet resulted in a reduction in mitochondrial fission potential and a normalized or an enhanced expression of mitochondrial fusion-associated genes. Moreover, while the expression of PGC-1*α* in obese mice was reduced when compared to lean mice, the levels of PGC-1*α* in HF diet fed mice treated with an EET-agonist were higher than in both lean mice and mice fed a HF diet alone. Importantly, global knockdown of PGC-1*α* in mice fed a HF diet nullified the EET-A-mediated beneficial effects on mitochondrial fusion and ROS suppressing potential in visceral adipose tissue. Increasing HF intake has been shown to enhance free fatty acid generation and increase mitochondrial dysfunction and ROS [35–38]. Together, these results clearly indicate that a recruitment of PGC-1*α* is crucial to the beneficial effects of the EET-agonist on mitochondrial function and on the reduction of fission and increase of fusion-associated processes in both adipose and hepatic tissues.

Our observation linking EETs to mitochondrial viability and fusion potential is supported by the demonstration that EETs protect neurons from oxidative damage while stimulating mitochondrial biogenesis [39]. Approximately 90% of cellular ROS are generated by the mitochondria and contribute to the mitochondrial energy metabolism [23]. SIRT3 is important in ROS suppression, mitochondrial biogenesis, and metabolic homeostasis, including mitochondrial fatty acid oxidation, and the target of PGC-1*α* [23, 24]. Multiple studies have reported the importance of mitochondrial function and dynamics in health and aging; for review see [28]. Knockout mice of Mfn1, Mfn2, and OPA1 are all embryonic lethal [40] and mutations in OPA1 in humans are associated with hereditary blindness, while Mfn2 mutations are the cause of Charcot-Marie-Tooth disease [27, 40, 41]. Since mitochondrial integrity encompasses the balance between mitochondrial fusion and fission [42, 43], an increase of Mfn1 and Mfn2 or decrease in fission DRP1 and fis is an indication of mitochondrial integrity and less oxidative stress that is responsible for mitochondrial damage and subsequent clearance by mitophagy [44].

This is in agreement with our data that showed liver-specific knockout of Mfn2 in mice is linked to impaired glucose metabolism, to decreases in SIRT1, SIRT3, and insulin signaling resulting in hepatic steatosis [45]. Thus, we show a PGC-1*α*-dependent increase of HO-1 expression, thereby potentiating the beneficial effect of PGC-1*α* itself. These results are supported by the demonstration that doxorubicin-induced oxidative stress increased mitochondrial fragmentation and Fis1 expression, causing mitophagy and mitochondrial fragmentation, an effect that was prevented by increased levels of HO-1 favoring expression of Mfn1 and Mfn2 [46].

Furthermore, we report that short- and long-term treatment with EET-A normalized impaired insulin receptor phosphorylation and decreased fasting blood glucose, BW, VO_2_, and RQ in mice fed a HF diet. The increase in VO_2_ may be related to an EET-mediated alternate metabolic strategy of HF-fed mice, that is, to increase the metabolic rate in response to increased EET-A-mediated PGC-1*α* expression and mitochondrial function leading to a reprogramming of adipose stem cells to brown-like cells, thus allowing the recovery of homeostatic control and the subsequent decrease in body fat, increase in insulin sensitivity, and normalization of oxygen consumption. Further, in agreement with our results an increase of PGC-1*α* is associated with the mechanism controlling mitochondrial biogenesis and integrity [47] and increased insulin sensitivity [16]. Increase of insulin receptor phosphorylation as a result of activation of HO-1 gene expression by EET-agonist is responsible for control of adiposity and insulin sensitivity (reviewed in [18, 48]).

This supports the hypotheses linking EET-A, the increase of PGC-1*α*, mitochondrial fusion, SIRT1, pAMPK, and HO-1 expression to a metabolic adipocyte reprogramming that may influence adipocyte hemostasis and delay terminal differentiation as evidenced by a decrease in Peg1/MEST and an increase in insulin receptor phosphorylation and adiponectin downstream signaling, including pAMPK. AMPK is as a master regulator of energy expenditure and performs its actions based on changes in the intracellular AMP/ATP ratio [49]. In metabolic organs, such as the liver, adipose tissues, and skeletal muscle, activated AMPK stimulates catabolic processes, while inhibiting anabolic processes [50]. As AMPK through its energy expenditure regulation also impacts the cellular redox balance of NAD^+^ and NADPH it can also activate SIRT1 deacetylation-activity, which is dependent on cellular NAD^+^ levels [51]; meanwhile several actions of AMPK have been shown to be dependent on SIRT1 activity [52]. AMPK and SIRT1 regulate PGC-1*α* activity through phosphorylation and deacetylation, respectively, and together AMPK, SIRT1, and PGC-1*α* control a wide range of metabolic processes, including insulin receptor signaling and gluconeogenesis in the liver, adipocyte browning and thermogenesis, and mitochondrial biogenesis [50, 51, 53]. EET-A increased pAMPK and SIRT1 levels in both adipose and liver tissues of HF diet fed mice corroborating the EET-mediated increase in total body oxygen consumption.

The importance of PGC-1*α* in adipose tissue has been established in mice lacking PGC-1*α* specifically in adipocytes which develop insulin resistance and have increased circulating lipid levels when fed a HF diet [16]. PGC-1*α* control of energy homeostasis suggests a role as a target for both antiobesity and diabetes drugs [54]. The finding that EET-A restored oxygen consumption and respiratory quotient to levels found in lean mice is supported by the beneficial increase in PGC-1*α*, SIRT1, pAMPK, and COX-IV (a unit of the mitochondrial OXPHOS complex) in the adipose tissue of mice fed a HF diet and treated with EET-A, thus highlighting the role of EETs in mitochondrial energy production and expenditure. EET-A increased PGC-1*α* expression in both adipose and hepatic tissue of mice fed a HF diet as compared to obese mice. PGC-1*α* is abundantly expressed in tissues with high energy demand [13, 47], and its induction, along with AMPK and SIRT1, regulates mitochondrial oxidative metabolism as well as mitochondrial biogenesis [52, 53, 55]. Thus, PGC-1*α* is critical in the activation of mitochondrial function including quality control, fusion potential, thermogenesis, and energy expenditure.

PGC-1*α*, UCP1, TFAM, and HO-1 levels were increased in adipose tissue of mice treated with EET-A, thus signifying a role of EETs in thermogenesis and browning of adipose tissue, which is masterly regulated via PGC-1*α* activation [52, 53, 56]. EET-mediated induction of PGC-1*α* and HO-1 favors an increase in mitochondrial fusion, biogenesis, and OXPHOS, thereby providing metabolic protection in HF diet induced obesity in mice; this finding is supported by recent reports by us and others showing that HO-1 regulates mitochondrial function, quality control, biogenesis, and dynamics [46, 57, 58].

Regardless of the duration of treatment, EET-A reduced HF diet induced weight gain while normalizing blood glucose levels, thus confirming the beneficial EET-mediated effects in diet-induced obesity [8, 11, 59]. The restoration of the metabolic parameters was mirrored by the normalization of the adipogenic markers FAS, aP2, PEG1/MEST, and C/EBP*α*, as well as the reduction of inflammation (TNF*α* and IL-6) in visceral fat obtained from EET-A-treated mice fed a HF diet as compared to the obese control mice. The normalization of adipogenic and inflammatory markers in HF diet fed mice treated with EET-A was coupled with increased levels of adiponectin and HO-1, both key to the maintenance of adipocyte maturation in favor of early-stage adipocyte differentiation, that is, healthy adipocytes [8, 58, 60–62].

The present study is of substantial interest from both a basic and a clinical science perspective, defining the existence of an EET-PGC-1*α*-HO-1 regulatory axis that can ameliorate the deleterious effects of HF diet-induced metabolic abnormalities, including insulin resistance, and prediabetic conditions involving adipose tissues. A deeper understanding of the mode of action of EET-PGC-1*α* axis necessary to maintain mitochondrial dynamics by favoring fusion over fission, increased insulin sensitivity, and improved energy expenditure will, we believe, provide new approaches in the treatment of obesity and metabolic syndrome associated complications, including insulin resistance, and subsequent cardiovascular complications. We consider the metabolically favorable interaction of lipid mediators generated by CYP450 epoxygenase that provide the potential of therapeutic targeting of the mitochondria dynamics in order to increase fusion over fission potential and other mitochondrial associated parameters that are dependent upon PGC-1*α* expression. This approach offers a portal to the treatment of metabolic diseases and hypertension with the result of an improved outcome in what is acknowledged to be a global epidemic of obesity.

## Figures and Tables

**(a):** 

Variable	Lean	HF	HF+EET-A	HF+EET-A+Ln PGC-1*α*(sh)
BW (g)	25.8 ± 0.35	30.2 ± 0.85^**∗**^	23.9 ± 0.19^#^	29.0 ± 1.47^##^
FBS (mg/dl)	107.5 ± 4.84	209.5 ± 9.84^**∗**^	156.0 ± 11.06^#^	263.5 ± 21.71^##^
BP (mm Hg)	104.0 ± 1.34	148.2 ± 3.01^**∗**^	1.05 ± 1.11^#^	137.0 ± 2.33^##^
VO_2_ (ml/kg/min)	53.92 ± 2.82	45.54 ± 1.82^**∗**^	77.17 ± 3.38^#^	46.46 ± 3.72^##^
RQ (CO_2_/O_2_)	0.74 ± 0.003	0.77 ± 0.007^**∗**^	0.74 ± 0.01^#^	0.78 ± 0.003^##^

Values are means ± SEM of 6 mice per group. Differences at *p* < 0.05 were considered significant. ^*∗*^ *p* < 0.05 versus lean, ^#^ *p* < 0.05 versus HF, and ^##^ *p* < 0.05 versus HF+EET-A. Body weight (BW). Blood pressure (BP). Fasting blood sugar (FBS). Oxygen consumption (VO_2_). Respiratory quotient (RQ).

**(b):** 

Variable	Lean	HF	HF+EET-A
BW (g)	28.68 ± 1.21	56.63 ± 0.88^**∗**^	32.43 ± 0.83^#^
FBS (mg/dl)	109.5 ± 4.84	286 ± 8.11^**∗**^	100.2 ± 11.32^#^
BP (mm Hg)	105.8 ± 2.87	156.7 ± 6.87^**∗**^	113.5 ± 4.44^#^
VO_2_ (ml/kg/min)	56.2 ± 2.65	33.77 ± 1.19^**∗**^	57.95 ± 3.12^#^
RQ (CO_2_/O_2_)	0.73 ± 0.006	0.8 ± 0.011^**∗**^	0.75 ± 0.007^#^

Values are means ± SEM of 3–5 mice per group. Differences at *p* < 0.05 were considered significant. ^*∗*^ *p* < 0.05 versus lean; ^#^ *p* < 0.05 versus HF. Body weight (BW). Fasting blood sugar (FBS). Blood pressure (BP). Oxygen consumption (VO_2_). Respiratory quotient (RQ).
